# Visual impairment in multiple sclerosis involving post-geniculate gray and white matter: above and beyond

**DOI:** 10.3389/fneur.2026.1875359

**Published:** 2026-08-28

**Authors:** Xiaojun Zhang, Gordon T Plant

**Affiliations:** 1 Department of Neurology, Department of Ophthalmology and Visual Science, The Ohio State University College of Medicine, Wexner Medical Center, Columbus, OH, United States; 2 Department of Translational Neuroscience and Stroke, University College London, London, United Kingdom

**Keywords:** cognitive impairment, cortical atrophy, MRI, multiple scleorsis, ocular motor, optical coherance tomography, post-geniculate visual pathway, vision

## Abstract

Visual impairment is one of the most common and disabling manifestations of multiple sclerosis (MS). While visual dysfunction related to optic neuritis and brainstem involvement is well recognized, visual impairment associated with post-geniculate visual pathways—including the optic radiations and visual cortex—remains comparatively under-studied and under-recognized in clinical practice. Increasing evidence from advanced neuroimaging, optical coherence tomography, and neuropsychological testing has demonstrated that lesions affecting post-geniculate white and gray matter contribute to both symptomatic and subclinical visual dysfunction in people with multiple sclerosis (PwMS). These abnormalities include homonymous visual field defects, visuoperceptual dysfunction, visual cognitive impairment, and higher-order ocular motor abnormalities. Cortical lesions and cortical thinning correlate with deficits in visual memory, visuospatial processing, and processing speed, while optic radiation damage contributes to retrograde transsynaptic degeneration and impaired visual information processing. Emerging eye-movement paradigms further suggest that higher-order ocular motor dysfunction reflects widespread gray matter involvement and may serve as a sensitive biomarker of disease burden. This review summarizes current histopathological and imaging evidence for post-geniculate pathway involvement in MS and discusses its clinical relevance for diagnosis, monitoring, and functional assessment.

## 1 Introduction

Visual symptoms and related disability are very common in patients with multiple sclerosis (PwMS). Furthermore, vision is rated by PwMS as their second most significant loss of function after walking (1). There is a tendency, given the signal and frequent occurrence of optic neuritis, to overlook the more central visual pathology in MS. Acute lesions in the chiasm and optic tracts are unusual but do occur, as do lesions, particularly of the large tumefactive phenotype, affecting the optic radiation and visual cortex. We also must consider disability resulting from involvement of ocular motor cortex, of the corona radiata, brainstem, cerebellum and cranial nerves subserving oculomotor control. Understanding how to localize visual disturbances in MS is germane to detecting both clinical and subclinical manifestations of disease activity that influence management. Optic neuritis is well known to be the most common early visual manifestation of MS. Diplopia and nystagmus related to brainstem and/or cerebellar lesions are also well known to be major causes of visual disability. On the other hand, visual impairment in PwMS associated with post-geniculate gray and white matter involvement, affecting either or both the afferent and efferent visual pathways, has been less studied. In recent years, the advancements in structural and functional measures of afferent and efferent visual pathway integrity have provided more information in this area, prompting our understanding about the gamut of underlying pathogenic mechanisms of vision related to disability in this condition (2). In this review, we will not discuss the visual symptoms related to demyelination in the anterior visual pathways, such as acute monocular vision loss and the Uhthoff and Pulfirch phenomena but will focus on visual impairment related to post-geniculate white and gray matter dysfunction in PwMS.

### 1.1 Literature search strategy

A narrative literature review was conducted using PubMed and Google Scholar databases to identify relevant articles published in English through May 2026. Search terms included combinations of “multiple sclerosis,” “optic neuritis,” “visual pathway,” “retina,” “optical coherence tomography,” “retinal nerve fiber layer,” “ganglion cell-inner plexiform layer,” “MRI,” “brain atrophy,” “white matter lesions,” “optic radiation,” “post-geniculate visual pathway,” “visual cortex,” “cortical lesions,” “higher-order visual dysfunction,” “ocular motor dysfunction,” “internuclear ophthalmoplegia,” “eye movement abnormalities,” “cognition,” and “cognitive impairment.” Additional studies were identified through manual review of references cited in relevant publications. The literature search encompassed publications available from database inception through December 2025. Original studies, systematic reviews, meta-analyses, and consensus statements addressing structural, functional, and neuroimaging correlates of visual dysfunction in multiple sclerosis were prioritized. Studies were selected based on their relevance to the scope of this review and their contribution to current understanding of visual pathway involvement in multiple sclerosis. Articles not directly related to visual dysfunction in multiple sclerosis, non-English language publications, and studies lacking sufficient clinical or imaging data were generally excluded. Case reports and small case series were included selectively when they provided unique insights into uncommon manifestations or emerging concepts.

### 1.2 Histopathological and image findings of post-geniculate gray and white matter involvement in patients with MS

MS has historically been defined as a chronic inflammatory disease of the central nervous system (CNS), which leads to focal lesions in the white matter of the brain and spinal cord, characterized by primary demyelination with a variable extent of axonal loss. In the past MS pathology was centered on the focal demyelinated plaques in the white matter but it has become clear that lesions are also present in the gray matter, including the cortex, basal ganglia, brain stem, and the gray matter of the spinal cord. Furthermore, there is neurodegeneration giving rise to axonal loss in normal appearing white matter and diffuse neurodegeneration in the entire gray matter. These changes result in profound atrophy, which develops particularly in the progressive stage of the disease (3). With modern techniques such as immunohistochemical staining and Magnetic Resonance Imaging (MRI), our understanding of the details of that pathology, and especially how it evolves over time, has been revolutionized. Lesions involve not only potentially all white matter (with a predilection for periventricular and juxtacortical areas, optic nerve and spinal cord) but also subpial and leptomeningeal cortex, the thalamus, pons, and retina (4).

MRI studies of white matter lesions that meet diagnostic criteria for dissemination in time and space have proven to be important biomarkers of MS, not only playing key roles in diagnosis and management, but also aiding our understanding of the underlying pathological processes and mechanisms related to neurological disability in MS. More recently, with the application of 7.0 Tesla ultra-high-field MRI, researchers have focused on finding novel and more specific imaging biomarkers to enhance diagnostic accuracy and reduce misdiagnosis. Among these, the central vein sign (CVS), cortical lesions (CL), and the paramagnetic rim lesion (PRL) have gained considerable attention in the past 2 decades (5). These advanced image biomarkers have demonstrated increased specificity for the differential diagnosis of MS and have been incorporated into the evolving diagnostic framework, including the 2024 revisions to the McDonald criteria, in which CVS and PRL are recognized as supportive imaging features that may improve diagnostic confidence (6). Furthermore, studies have shown that CL and PRL may serve as prognostic biomarkers, with CL correlating with cognitive impairment and PRL being associated with chronic active inflammation and early disability progression. (7). In addition to these biomarkers, certain MRI phenotypes, including tumefactive demyelinating lesions and the concentric ring pattern characteristic of Balo concentric sclerosis, may indicate more aggressive or atypical disease presentations and can pose important diagnostic and therapeutic challenges.

### 1.3 Post-geniculate white matter lesions in Patients with MS and related visual impairment

#### 1.3.1 Optic radiation (OR) lesions in PwMS and related visual impairment

Optic radiation lesions are particularly common in MS, both at autopsy and in imaging studies. A postmortem study of 20 cases of MS found lesions in optic radiation in 85% of patients (8). In a subsequent autopsy study of 22 cases of MS, plaques were most commonly periventricular, the regions most affected being the lateral angles of the anterior horns and bodies and around the inferior and posterior horns. These findings have been confirmed in MRI studies of the brain in MS during life where evidence of pathology affecting the optic radiation is also common (9). In a study of 114 cases of MS, the peritrigonal white matter was involved in 90% of cases and the white matter around the occipital horns in 83% of cases (10). Furthermore, a study also found evidence on MRI of asymptomatic lesions affecting the optic radiation in 20 of 28 cases of clinically isolated optic neuritis (1, 11). Despite the above, visual symptoms and visual function impairment related to retro-chiasmal, in particular OR, lesions have been considered as very uncommon.

In the 1990's Plant et al. (12) reported 18 MS patients who presented with symptomatic homonymous hemianopic visual field defects (VFD) due to retro-chiasmal lesions during the course of their disease using Goldmann perimetry. The lesions responsible involved the posterior OR in eight cases, the optic tract and LGN in six cases, and the posterior limb of the internal capsule in 3 cases. The prognosis for recovery of the field defect was good; complete recovery occurred in 14 patients, and only two showed no recovery at all. The authors noted the striking characteristic of the post-geniculate lesions causing homonymous VFD is that they are usually very large, now designated as tumefactive MS. Indeed, it was pointed out that in the early imaging studies of MS cases using CT scanning most of the lesions (large enough to be visible on CT) presented with either hemianopia or hemiparesis. The authors discussed the mechanism of the discrepancy between the rarity of clinical evidence of impaired visual function and frequency of OR lesions on MRI, proposing that the more common small MS lesions affect proportionally a small volume of the OR (in comparison with a similar sized lesion in the optic nerve or optic tract) thus not causing detectable visual impairment. In tumefactive MS, the large OR lesions with accompanying oedema affect larger proportion of visual pathway hence causing symptomatic VFD. It is also the case that the degree of retinotopic integrity is important. In the optic tract retinotopy is intact and contained within a small volume in the coronal plane. However, between the LGN and striate cortex retinotopy becomes increasingly disordered and covers a much larger area in the coronal plane. Hence an MS lesion below a certain size will cause visual field loss that is characterized by affecting a relatively small visual field area and being patchier within that area.

By applying more advanced MRI with designed algorithmic and manual segmentation, a more recent study (13) investigated 34 relapsing remitting MS (RRMS) patients and found that OR lesion volume was correlated with a reduction in LGN volume compared to a healthy control group. In addition, LGN volume was not correlated with performance on low contrast visual acuity charts but was related to impairment of color vision as measured by Hardy-Randy-Ritter (HRR) test plates. The author suggested that LGN volume loss in MS indicates structural damage with potential visual function relevance, with direct retrograde degeneration from damage to the OR as the underlying cause. There will be a contribution from retrograde transsynaptic degeneration, but this will not involve additional loss of cell bodies in the LGN which will be the major contribution to the loss of volume.

Optic coherence tomography (OCT) is a high-resolution imaging technique suitable for sophisticated postprocessing. Measurements of retinal nerve fiber layer (RNFL) and combined ganglion cell and internal plexiform layer (GCIPL) thickness has been well-studied in MS and other conditions (14, 15), and has served as a structural retinal biomarker for early recognition and monitoring of inflammation and neurodegeneration in multiple sclerosis. Al-Louzi et al reported a case series of six cases where homonymous, hemi-macular GCIP thickness reduction was seen in conjunction with posterior visual pathway isolated occipital or post-geniculate lesions (16). Ilardi et al. (17) investigated macular GCIPL thinning in 135 patients with CNS demyelinating disorders, the majority being cases of relapsing remitting (RRMS) although some cases of progressive MS (PMS) were included. Homonymous hemi-macular thinning (a marker of retrograde direct or retrograde transsynaptic degeneration in the post-chiasmal visual pathway) was observed using OCT in only about 4% of MS patients. The rarity of this finding suggests that post chiasmal and post geniculate white matter lesions rarely result in retrograde degeneration, a significant contrast to the near universal finding of GCIPL thinning following optic neuritis (also direct retrograde degeneration) and transsynaptic retrograde degeneration in retro-chiasmal lesions due to stroke. Patients with this pattern of GCIPL often fail to complain of hemifield visual loss. This study further supports the previous findings by Plant et al regarding the rarity of symptomatic visual function impairment caused by OR lesions in MS. It should be noted that, (although relatively uncommon in MS, certainly in comparison with NMO) chiasmal and optic tract lesions are occasionally seen presenting with symptomatic visual field loss. However, more subclinical retinal neuroaxonal damage with retrograde trans-synaptic neuroaxonal degeneration has been found by OCT, showing GCIPL thinning related to OR lesions, and the severity of GCIPL thinning is correlated with both OR lesion volume and brain atrophy (18). While it may be rare for typical (non-tumefactive) lesions to cause a symptomatic relapse, the accumulation of demyelination due to OR lesions in MS may well be significant in contributing to visual processing speed impairment which is the most commonly affected cognitive feature in MS (19).

Although the cohort studied by Ilardi et al. (17) also included patients with AQP4-NMOSD and MOGAD, the small number of non-MS cases precluded meaningful comparisons among demyelinating disorders. Interestingly, trans-synaptic degeneration has also been demonstrated in MOGAD. In a cohort of 34 MOGAD patients, including 24 cases with previous history of optic neuritis and 10 cases without previous history of optic neuritis, Bollo et al. (20) found that retinal thinning was primarily driven by prior optic neuritis and correlated with reduced lateral geniculate nucleus volume and occipital cortical thickness, suggesting that neurodegeneration in MOGAD is closely linked to damage along the affected visual pathway. However, because retinal changes were largely determined by optic neuritis burden and no direct comparisons with MS were performed. These findings do not clarify whether retrograde transsynaptic degeneration is more prevalent or more severe in MOGAD than in MS. Nevertheless, direct comparisons among MS, MOGAD, and AQP4-NMOSD remain limited, and further studies are needed to determine whether the frequency and severity of retrograde transsynaptic degeneration differ among these disorders.

The relatively low prevalence of homonymous hemi-macular GCIPL thinning in MS compared with ischaemic stroke likely reflects several factors. Rather than lesion size alone, the key distinction is the degree to which a lesion produces a discrete, retinotopically organized interruption of the post-geniculate visual pathway. Even small infarcts involving strategic portions of the lateral geniculate nucleus, optic radiations, or occipital cortex may produce a congruous homonymous visual field defect and subsequent retrograde transsynaptic degeneration (21). In contrast, MS lesions affecting the optic radiation are often multifocal, partial, temporally dispersed, and accompanied by diffuse neurodegeneration, making it less likely that they will produce a sharply demarcated homonymous hemi-macular GCIPL pattern. In addition, retinal neuroaxonal loss in MS is frequently multifactorial, arising from prior optic neuritis, subclinical optic nerve involvement, diffuse neurodegeneration, ageing, and microvascular comorbidities, which may obscure the characteristic homonymous pattern of hemi-macular thinning. Consequently, although subclinical retrograde transsynaptic degeneration can occur following optic radiation lesions in MS, the classic OCT appearance of homonymous hemi-macular GCIPL thinning is much less common than after focal post-chiasmal lesions such as stroke.

Despite the growing utility of OCT in detecting retinal changes associated with retrograde transsynaptic degeneration in MS, accurate interpretation requires careful consideration of several technical and clinical challenges. Segmentation errors, and poor image quality may affect GCIPL measurements and mimic or mask hemi-macular thinning. Differentiating retrograde transsynaptic degeneration from primary retinal pathology or direct retrograde degeneration due to optic nerve involvement may be difficult, particularly in patients with previous optic neuritis. Coexisting glaucoma, epiretinal membrane, high myopia, segmentation errors, and poor image quality may affect GCIPL measurements and mimic or mask hemi-macular thinning. Careful correlation of visual field testing with MRI localization of post-geniculate lesions is, therefore. essential. The presence of diffuse bilateral retinal thinning, common in MS, may further reduce the conspicuity of homonymous patterns, emphasizing the importance of topographic analysis and longitudinal assessment rather than relying solely on absolute thickness measurements.

### 1.4 Higher order visual dysfunction and cortical lesions in patients with MS

Higer order visual dysfunction refers to symptoms and signs resulting from disorders affecting specialized visual cortical regions and their underlying white matter. White matter anatomical and functional features that need to be considered are input, output and lateral connections. Functional specialization for distinct visual features defines higher-order visual regions and their corresponding networks. Two major pathways extend from occipital striate cortex, with one projecting to the parietal lobes, referred to as the “where” pathway, and one to the temporal lobes, referred to as the “what” pathway (21, 22). Purely cortical lesions affecting these regions do not result in scotomata and instead result in impairment of submodalities of visual processing such as the perception of motion, visual-spatial relationships, faces, colors, or objects (23, 24). In cases due to stroke or other focal pathology homonymous visual field defects are common but are due to damage to the underlying white matter, the optic radiation. Furthermore, to be symptomatic most cases are due to bilateral symmetrical lesions as unilateral lesions giving rise to, for example, hemiachromatopsia (25) or hemiakinetopsia (26) may be asymptomatic. Conditions causing higher order visual dysfunction are broad, of which stroke and neurodegenerative disorders are two of the most common neurological disorders in the older population, however it is relatively rarely reported in PwMS.

#### 1.4.1 Visual perceptual dysfunction in MS

Vision is fundamental to many aspects of human cognition, but there are few studies of higher order visual dysfunction in PwMS, the most recognized being visuo-perceptive dysfunction, which is believed probably to be due to a combination of damage to the afferent visual pathways and to primary visual cortex and beyond (27). In the study of an unselected series involving 49 MS patients with a mean estimated disability status scale (EDSS) score of 5.93, Vleugels et al. (28) reported an estimated failure of four or more visuo-perceptive function tests in 26% of PwMS. They found that among the 31 tests carried out, color discrimination (FM100 test); the perception of the Muller-Lyer illusion; the Visual Object and Space Perception battery (VOSP) (29); Object Decision; and the Birmingham Object Recognition Battery (BORB); (30) were the five most frequently failed tasks in PwMS. In addition to visuospatial perception, other vision related cognitive functions such as immediate and delayed visual memory, along with processing speed, were reported as the most commonly impaired cognitive domains in PwMS (31). Consistent with these findings, Brandstadter et al. (32) reported that word-finding difficulty is a prevalent deficit even in early MS, with impaired performance on rapid automatized naming (RAN) tasks reflecting slowed visual processing speed and inefficient integration of visual and language networks. It is noteworthy that the Facial Recognition of Famous People and Facial Recognition Test batteries which are commonly impaired in both congenital and acquired neurodegenerative disorders, were found to be intact in this group of PwMS (33).

#### 1.4.2 Other instances of vision related cognitive impairment (CI)

Other instances of vision related cognitive impairment (CI) have also been reported but these are rare and unusual cases. In 1987, Day et al. reported a 34-year-old woman with relapsing remitting MS (RRMS) who presented with alexia with agraphia and Gerstmann's syndrome as her third clinical event of MS. In this case, alexia for letters, words, sentences, clocks and music developed acutely in association with agraphia and dysphasia, Alexia persisted after resolution of the dysphasia before recovering completely. CT head revealed a large enhancing lesion at left temporal-parietal white matter extending to occipital horn of lateral ventricle. The authors considered that the responsible lesion involved the cortex and deep white matter of the left parieto-occipital region interrupting the connections of the visual cortex with Wernicke's area and the angular gyrus (34). In 2004, Mao-Draayer et al. reported a case of a 48-year-old man who presented with alexia without agraphia associated with right homonymous hemianopia as the initial manifestations of MS. He was able to name objects, letters, colors and numbers correctly, but was unable to read complete words or sentences despite being able to write fluently to dictation or spontaneously. MRI brain showed a non-enhancing lesion in the left occipital white matter adjacent to the posterior horn of the lateral ventricle, and an enhancing lesion in the splenium of the corpus callosum, which is a disconnection syndrome most commonly seen in stroke patients, where visual information from the right occipital cortex is prevented from reaching the intact left angular gyrus because of a lesion in the corpus callosum or adjacent parieto-occipital WM (35). Notwithstanding that vision related cognitive dysfunctions are rare in MS, it is important to keep in mind that they can be the initial presenting symptoms of MS.

#### 1.4.3 Epilepsy and its visual manifestations

Epilepsy and its visual manifestations are rare and currently believed to be related to cortical lesion in PwMS. For context, the prevalence of epilepsy in the general population is approximately 1%, whereas pooled estimates suggest a prevalence of 3.09% and an incidence of 2.28% in PwMS (36, 37). The types of epileptiform seizures are also varied but most reported are focal motor or sensory seizures, developing into secondary generalized seizure (38). Disturbances of color vision and visual hallucinations may be symptoms of a rare form of epilepsy in PwMS. Compared to other CNS demyelinating disorders such as myelin oligodendrocyte glycoprotein antibody-associated disease (MOGAD), in which the reported seizure prevalence is 5%−21% (39, 40), seizure is relatively rare, however, because seizure is often an isolated first manifestation of MS, for patients presenting with episodic visual disturbance, MS should be kept on the differential diagnosis along with other more common causes of occipital seizure.

#### 1.4.4 Structure function correlation between MS cortical lesions and visual dysfunction

To localize the structural lesion that causes function change has always played an important role to help us better understand and provide better care for patients not only with MS, but for all the patients with neurological conditions. Although initially considered as a white matter disease, gray matter cortical lesions (CLs) were found to be abundant histologically by post-mortem also by more advanced neuroimaging in PwMS in the modern era (3–58). Neuroimaging correlates of cognitive disorders in PwMS evolved further at the turn of 21st century, helping us to better understand the connections between brain injury and performance on cognitive batteries (41). As cortical lesions identified by ultra-high-field MRI seem highly specific for MS, they have become an integral part of the diagnostic criteria for the disorder in the most recently updated diagnostic criteria (6). Earlier studies found that total MS lesion area and whole brain atrophy are related to cognitive dysfunction including visuospatial problem solving. More recent studies have further demonstrated that cortical lesions and cortical atrophy better correlate with cognitive impairment compared to global atrophy and WM lesion measures. Indeed, it has become clear that cortical gray matter damage is the most specific structural defect underlying cognitive impairment in MS (42, 43).

#### 1.4.5 Focal cortical lesion correlated cognitive impairment (CI) and visual dysfunction

Recent studies using ultra-high-field MRI have demonstrated a clear correlation between focal cortical lesions and CI (44). Furthermore, Harrison et al. (45) have described a number of morphological classes of cortical lesion which are differentially related to cognitive impairment. They report that leukocortical CLs had the greatest effect on cognition. In their complex cognitive testing battery, brief visuospatial memory testing (BVMT) score in PwMS was lower than the healthy controls and significantly correlated with count and volume of CLs. Durozard et al. (46) highlight that focal CLs, although found in all stages of MS, are especially prevalent at the early stages, confirming that the leukocortical lesions more effectively accounted for cognitive deficits. Considering the above diverse but significant studies concerning vision-related cognitive function impairment correlated with focal cortical lesions in PwMs, we can ask the question: how much, if any, vision related cognitive impairment recovers spontaneously or after treatment, especially in relapsing remitting MS (RRMS)? To date, no research concerning recovery of residual cognitive function correlated to focal cortical lesions has been published.

#### 1.4.6 Cortical atrophy or thinning correlated with CI and visual function

Cortical atrophy has been found in different phenotypes of MS including early-stage MS. In 2016, Tillema et al. (47) reported a very interesting study that explored MRI correlates between regional cerebral cortical thinning and global CI as well as impairment in specific cognitive domains. In this multicenter study conducted in seven European centers of the Magnetic Resonance Imaging in MS (MAGNIMS) network, the authors found that in addition to mild global cortical thinning in RRMS, temporal lobe thinning is more common in cognitively impaired compared to cognitively preserved PwMS. Of interest, the study also found Verbal memory scores to be correlated to regional cortical thinning in the insula, whereas visual memory scores correlated to parietal thinning. Around the same time, Pitteri et al. (48) conducted a retrospective longitudinal study to investigate the role of CI, including visuospatial learning, visual information processing speed and attention functioning in a study of the earlier identification of RRMS patients with a higher risk of disease/disability progression. This study included 78 patients with RRMS who completed neuropsychological examination and structural MRI at the time of diagnosis. Each patient was clinically evaluated every 6 months, and cortical thickness was quantified at baseline and at the end of the follow-up. They found that CI at the time of diagnosis was a good predictor of conversion to definite MS, of disability progression, also of transition to a secondary progressive phase and of cortical thinning (*p* < 0.001). Another multi-center study published in 2021 further investigated both the longitudinal cortical thinning progression and different cortical thinning patterns in different MS phenotypes (49). They found baseline frontal, parietal, and sensorimotor atrophy driven by RRMS, while PMS showed additional parietal, insular and sensorimotor cortical atrophy. At 1-year follow up, additional frontal and temporal cortical thinning was detected in RRMS patients, while a widespread cortical thinning was found in PMS patients. In their discussion of clinical relevance of cortical thinning, they specified that the involvement of the lateral orbitofrontal cortex and the inferior parietal lobule have been correlated with associative dysfunction, ideomotor apraxia, sensory agnosia, and visual disturbances related to tactile visual perception and construction of object shapes (50) which impair clinical performance. The association between visual pathway abnormalities and cerebral neurodegeneration extends beyond adult-onset MS. In a recent paediatric-onset MS (PoMS) cohort, Sosa et al. (51) performed a cross-sectional study with retrospective ascertainment of patients with PoMS evaluated at a single center with expertise in PoMS and Neuro-Ophthalmology. They reported among 61 patients (122 eyes) with PoMS that pRNFL thinning was associated with reduced whole-brain, total gray matter, and thalamic volumes, as well as increased lesion and black hole burden. Furthermore, impaired color vision and reduced high-contrast visual acuity were associated with smaller hippocampal volumes.

A study from China by Liu et al. (52) utilizing a 3.0-T MRI system and voxel-based morphology (VBM) analysis. Twenty-seven patients with RRMS were compared with 23 patients with neuromyelitis optica (NMO). A limited number of regions in visual cortex were significantly thinner in NMO patients than in a healthy control group. The MS patients, on the other hand, exhibited more widespread cortical thinning and significantly greater cortical thinning in the insula and the parahippocampus compared with NMO. The extent of cortical thinning in several brain regions correlated with cognitive measures in MS, but not in NMO. The authors thus suggest that the pathological substrate of impaired cognition in MS and NMO could be different, specifically that cognitive change is linked to cortical atrophy in MS, and the altered cognition in NMO may be correlated to widespread subtle abnormalities in white matter. This observation would be compatible with the observation that white matter lesions in NMO are less likely to show recovery, perhaps related to the greater likelihood of preserved axonal integrity and potential for remyelination in the case of MS lesions. More recently, a multicenter study comparing MOGAD, AQP4-IgG-positive NMOSD, and relapsing-remitting MS demonstrated distinct patterns of gray matter atrophy among these disorders. While gray matter loss was diffuse in RRMS, MOGAD showed preferential involvement of the temporal cortex and deep gray matter, whereas AQP4-NMOSD predominantly affected the occipital cortex, suggesting disease-specific patterns of neurodegeneration and tissue injury (53). These structural differences may also translate into distinct neurovisual phenotypes. Compared with the diffuse neurodegenerative changes and multifocal visual pathway involvement characteristic of MS, visual dysfunction in AQP4-IgG-positive NMOSD and MOGAD is predominantly driven by severe episodes of optic neuritis and associated retinal neuroaxonal loss. OCT studies have demonstrated more profound RNFL and GCIPL thinning following optic neuritis in NMOSD and MOGAD than in MS, whereas diffuse retinal thinning independent of optic neuritis and subtle retrograde transsynaptic degeneration appear to be more prominent features of MS (15, 20). Likewise, higher-order visual and ocular motor dysfunction in MS likely reflect widespread involvement of distributed cortical and white matter networks, whereas corresponding data in NMOSD and MOGAD remain relatively limited. The contribution of ocular motor dysfunction to visuo-cognitive impairment in MS is discussed separately in the following section.

### 1.5 Higer order ocular motor dysfunction and cognitive impairment in patients with MS

Eye movement deficits are common in PwMS, with typical and most well-known presentations as ocular misalignment causing diplopia and instability with nystagmus such as internuclear ophthalmoplegia (INO) related to brainstem or cerebellar white matter lesions (54). Ocular motor dysfunction reflects disruption of widespread brainstem, cerebellar, and cortical networks and has been proposed as a biomarker of cognitive dysfunction in MS (55). INO and other ocular motor abnormalities may also contribute to visuo-cognitive impairment in MS. Recently, Hof et al. (56) demonstrated that severe INO may independently impair performance on visually based cognitive tests such as the Symbol Digit Modalities Test and Concept Shifting Test, likely owing to the increased demands placed on rapid saccadic eye movements. In contrast, performance on the auditory Paced Auditory Serial Addition Test was unaffected. These findings suggest that ocular motor dysfunction may not only reflect cognitive impairment but also confound the interpretation of visual cognitive tests, emphasizing the need to consider eye movement abnormalities when assessing cognition in people with MS. In addition to INO, eye movement abnormalities involving saccades, smooth pursuit, fixation, and anti-saccade performance correlate with deficits in attention, information processing speed, and executive function (56). In addition to the bedside examination, new and patient-friendly techniques for recording eye movements at the bedside, such as vestibular evoked myogenic potentials (VEMPs) and head impulse testing with the VOG (VHIT) have been used to screen PwMS for vestibular dysfunction and help us to learn about the pathophysiology and localization of the pathology underlying their eye movement disorders (57). On the other hand, subtle ocular motor dysfunction in PwMS related to the higher order ocular motor control network may have been underrecognized, even though lately this has been considered as a potential biomarker for cognitive impairment of PwMS (58).

Here we focus on the ocular motor dysfunction related to gray matter CLs and related cognitive function impairment. Serra et al in their comprehensive review in 2018 (58) summarized some saccadic testing paradigms and their dysfunction in PwMS. First, the antisaccade task is the most widely used ocular motor test of cognitive control, a version of this test can be administered manually at the bedside. The dorsolateral prefrontal cortex (PFC) seems to have a primary role in the inhibition of automatic, reflexive saccades otherwise initiated by the parietal eye fields. MS patients make more errors in the antisaccade test and generate saccades with delayed latency compared with controls. Such poor performance in MS may correlate also with cerebellar dysfunction as the role of the cerebellum in cognitive control is increasingly recognized. Secondly, PwMS also make mistakes when required to generate saccades toward a remembered target (the so-called memory guided saccades) which is thought to reflect a deficit of working memory. During this task, their saccades are also inaccurate, especially when asked to execute a memorized sequence of target jumps. Finally, saccades made in response to predictable target jumps are usually hypometric in MS, and latencies are increased for saccades toward visual targets presented at random locations along with a visual distracter. These abnormalities also reflect inability to maintain inhibitory control through the PFC and its connections to thalamocorticostriatal circuits.

In 2019, Kincses et al. (59) reported a study of gray matter atrophy correlated with subclinical ocular motor deficits in PwMS. They include 34 clinically stable, treated RRMS patients with mild disability and 34 healthy controls. Group difference and correlation with clinical parameters were analyzed in case of the latency, peak-velocity, gain, disconjugacy index, and performance during a saccade and anti-saccade task. High-resolution T1 weighted, T2 FLAIR, and double inversion recovery images were acquired on 3T to evaluate the correlation between behavioral and MRI parameters, such as T2 lesion and T1 black-hole burden, global brain gray, and white matter atrophy. The results found the significantly increased latency in the prosaccade task and significantly worse performance in the anti-saccade task in PwMS. The peak velocity and latency of the anti-saccade movement correlated with the number of black holes, but none of the eye movement parameters were associated with the T2 lesion burden or location. Global gray matter volume correlated with saccade and anti-saccade latency, whereas white matter and total brain volume did not. Local gray matter atrophy in the left infero-parietal lobule and temporo-occipital junction correlated with anti-saccade peak velocity. The study concluded that neurodegeneration-like features of the MRI (black-hole, gray matter atrophy) are the best predictors of ocular motor deficits in MS. but none of the eye movement parameters were associated with the T2 lesion burden or location. Importantly, anti-saccade peak velocity correlates with gray matter atrophy in the left parietal regions, which are frequently implicated in attention tasks.

In 2023, Bijvank et al. (60) reported a single-center, cross-sectional cohort study that interrogated the structural and functional relationships of double-step saccades in multiple sclerosis. Data was collected for double-step saccades, cognitive function (extended Rao's Brief Repeatable Battery), disability (EDSS), and visual functioning in daily life (National Eye Institute Visual Function Questionnaire). MRI was used to quantify gray matter atrophy and multiple sclerosis lesion load. They included 209 PwMS with 63% RRMS and 60 age and gender matched healthy control subjects. The proportion of correct double-step saccades was significantly reduced in PwMS compared to controls. Consistent with this, there was a significantly larger double-step dysmetric saccadic error in PwMS compared to controls. Impaired double-step saccadic metrics were consistently associated with more severe global and local gray matter atrophy, lesion load, progressive phenotypes, more severe physical and cognitive impairment and visual functioning. The authors concluded that double-step saccades represent a robust metric that revealed a novel eye-movement impairment in PwMS. Double-step saccades outperformed other saccadic tasks in their statistical relationship with clinical, cognitive and visual functioning, as well as global and local gray matter atrophy. They suggested that double-step saccades should be evaluated longitudinally and tested as a potential novel outcome measure for remyelination trials in multiple sclerosis.

## 2 Conclusion

Although visual dysfunction in multiple sclerosis has traditionally been attributed to optic nerve and brainstem lesions, increasing evidence suggests that higher-order visual and ocular motor abnormalities related to optic radiation, cortical, and distributed network involvement are important yet underrecognized manifestations of the disease. Advances in optical coherence tomography, ultra-high-field MRI, volumetric MRI, eye movement recording, and cognitive assessment have revealed both clinical and subclinical dysfunction affecting the afferent and efferent visual systems. While rare manifestations such as homonymous hemianopia and alexia with or without agraphia may represent presenting features of MS, more subtle visuospatial, visuoperceptual, and ocular motor abnormalities are increasingly recognized and often correlate with specific cortical lesion phenotypes, gray matter atrophy and network dysfunction. Visuospatial and visuoperceptive disfunction have been found to be the most common cognitive deficits in PwMS Ocular motor abnormalities, particularly saccadic dysfunction, are closely linked to cognitive impairment and may provide objective markers of distributed brain injury. Together, these observations support a network-based model of visual dysfunction in MS that extends beyond the optic nerve and brainstem. Improved understanding of higher-order visual and ocular motor dysfunction may facilitate more accurate patient counseling and provide objective and reproducible outcome measures for future studies and therapeutic trials.
